# Impact of a smoke-free policy on smoking prevalence on hospital grounds: A before-after study

**DOI:** 10.18332/tpc/149476

**Published:** 2022-05-20

**Authors:** Heike H. Garritsen, Jentien M. Vermeulen, Andrea D. Rozema, Luc R. C. W. van Lonkhuijzen, Anton E. Kunst

**Affiliations:** 1 Department of Public and Occupational Health, Amsterdam UMC, University of Amsterdam, Amsterdam, The Netherlands; 2 Department of Psychiatry, Amsterdam UMC, University of Amsterdam, Amsterdam, The Netherlands; 3 Tranzo Scientific Center for Care and Wellbeing, Tilburg School of Social and Behavioral Sciences, Tilburg University, Tilburg, The Netherlands; 4 Department of Gynecologic Oncology, Amsterdam UMC, University of Amsterdam, Amsterdam, The Netherlands

**Keywords:** smoking, secondhand smoke, smoke-free policy, hospital

## Abstract

**INTRODUCTION:**

Studies on the impact of smoke-free policies (SFPs) on hospitals grounds on on-site smoking are scarce. On 1 October 2019, an SFP was implemented on the grounds of the Amsterdam UMC hospital in the Netherlands, including measures for sustained enforcement. This study assessed the impact of this SFP on smoking prevalence on hospital grounds up to 18 months after implementation.

**METHODS:**

Observations were systematically conducted 7 weeks before and after the SFP was implemented, and at 5 and 18 months afterwards. A total of 32 sites were included in the study, divided over two hospital locations. On each site, the number of smokers was systematically observed and categorized into staff, patient, student, or visitor. Smoking prevalence on hospital grounds was calculated by the number of observed smokers as a proportion of all people observed. Bubble maps were created to visualize changes in the geographical distribution of smokers.

**RESULTS:**

Smoking prevalence on hospital grounds decreased significantly from 17.4% before to 3.3% after implementation of the SFP. Following implementation, the largest decrease was observed in smoking among staff (-96.7%) and patients (-92.3%). The decrease in smoking prevalence was sustained 18 months after implementation (5.0%). The number of smokers decreased on nearly all sites.

**CONCLUSIONS:**

The substantial and sustained decrease in smoking prevalence found in this study highlights the potential of SFPs on hospital grounds to protect people from exposure to (secondhand) smoking. Continued enforcement of these SFPs seems essential to ensure ongoing compliance.

## INTRODUCTION

Killing more than 8 million people a year, the tobacco epidemic is one of the biggest public health threats the world has ever faced. Around 1.2 million of those deaths are the result of non-smokers being exposed to secondhand smoke (SHS)^1^. One of the most effective strategies to reduce SHS exposure is the implementation of smoke-free policies (SFPs)^2,3^. Besides protecting non-smokers from the involuntary inhalation of tobacco smoke, SFPs have also been proven to decrease smoking prevalence and foster smoking cessation attempts^4^.

As important and highly visible healthcare institutions, hospitals are often at the forefront of tobacco control and take the lead in implementing SFPs. SFPs in hospitals offer a healthy environment for staff, patients, and visitors, while delivering a public health message about the dangers of (secondhand) smoking^5^. Over the past decades, an increasing number of countries have prohibited smoking within hospitals^6^. More recently, hospitals have undertaken initiatives aimed at the implementation of totally smoke-free hospital sites, including the grounds outside the buildings^5^.

A number of studies have investigated SFPs on hospital grounds, with the majority of studies focusing on the impact of SFPs on self-reported smoking among staff. For example, employees of three hospitals reported reduced cigarette consumption^4,7,8^ and increased attempts to quit^4,8^ after implementation of an SFP. To date, however, field studies on the impact of SFPs on smoking on hospital grounds are scarce^9,10^. Such studies are important in order to determine to what extent people remain exposed to (secondhand) smoking. In 2012, a study in Australia found a significant reduction in on-site smoking behavior up to two years after implementation of an SFP^9^. More recently, a study in Rotterdam, the Netherlands, reported that the implementation of an SFP was associated with a substantial decrease in the number of observed smokers on hospital grounds^10^. However, the authors did not look at the long-term impact of the SFP.

Approximately 20.6% of adults in the Netherlands and 20.4% of adults in Amsterdam smoke^11,12^. On 1 October 2019, an SFP was implemented at the Amsterdam UMC, prohibiting smoking (including e-cigarettes) on all hospital grounds. The Amsterdam UMC is a Dutch academic hospital which consists of two locations: AMC and VUmc. The hospital has over 15000 employees and treats around 350000 patients each year. A comprehensive and multifaceted preparation preceded the implementation of the SFP. The board announced the SFP in their New Year’s speech and the implementation team started with weekly meetings by March. In over 50 meetings, stakeholders (e.g. policy makers, nurses, support staff) were informed about the upcoming policy, and potential barriers were identified and addressed. Furthermore, presentation on smoking (cessation) and the implementation of the SFP were held at clinical departments serving a patient population with a high smoking prevalence (e.g. surgery, psychiatry, cardiology, and pulmonology).

To increase support among stakeholders, the SFP aims were framed in a positive way: setting a good example, contributing to a healthy environment, and encouraging people to quit smoking. Smoking cessation support was available for staff in the weeks leading up to the SFP. They were offered nicotine replacement therapy and behavioral counseling through phone or group meetings, all free-of-charge. For patients, nicotine replacement and behavioral therapy was protocolled and made readily available to admitted patients who had to refrain from smoking during their admission. No smoking cessation support was offered to visitors. They were referred to their own general practitioner or a Dutch website offering guidance on where to find cessation counseling and support.

The SFP was communicated through letters sent to patients and included in communication with outside contractors. Banners, signs, and tiles, were placed to inform people about the SFP. All smoking facilities and ash bins were removed from the premises. After implementation, enforcement officers (two per hospital location) patrolled the grounds during office hours to inform people about the policy, answer questions, and ask anyone smoking on the grounds to put out their cigarette. Enforcement officers were hired by two professional organizations and received a one-hour training on their first working day. If any problems with regard to enforcement arose, security officers of both hospital locations could be asked to assist. To sustain the SFP after initial implementation, the policy was mentioned in the general invitation letters that patients received for their appointments as well as in the welcoming meeting for new employees. Signage was continued.

The aim of this study is to assess the impact of the SFP on smoking prevalence on hospital grounds. Using an observational before-after design, we assess both the short-term and long-term impact (up to 18 months) of the SFP.

## METHODS

### Procedure

On a map of both sub-locations, the grounds were divided into geographically discrete sites. Those sites were delineated by the natural borders of the location. A total of 32 sites were included in the study: 17 at location AMC and 15 at location VUmc.

Systematic observations were conducted between August and December 2019, from 7 weeks before until 7 weeks after the SFP was implemented. In addition, in order to determine long-term impact of the SFP, follow-up observations were conducted in February 2020 (n=2 observation rounds) and April 2021 (n=4 observation rounds). Both locations were visited once a week and all observations took place on weekdays between 11:30 and 15:00. This timeframe was chosen to cover lunch break, as it was expected that most people would smoke during this time. Observations were conducted by four researchers. The first author had experience with conducting observations and trained the other three researchers. This included explaining how long each site needed to be observed and how to distinguish the different categories of smokers. In an attempt to minimize intra-observer variability, the first observations were conducted together.

During an observation round, each site was visited once. The observer began at a predetermined starting point and walked along a predetermined route around the hospital. At each site, the observer counted the number of people in sight. Each observed individual was then categorized either as a smoker (holding a cigarette) or a non-smoker. Finally, smokers were categorized as either staff (e.g. wearing a uniform or hospital identification tag), patient (e.g. wearing a hospital gown or patient wrist band), student (e.g. present at the entrance of the faculty), or visitor (having none of these characteristics or if in doubt). As weather conditions may influence the extent of outdoor smoking on hospitals grounds^13^, we recorded the weather conditions for each observation period.

To determine the reliability of recorded observations of smoking, an inter-rater reliability assessment was conducted in two of the units during an earlier pilot study in which the first author (PW) was the gold standard observer for comparison with the study observer (BG).

### Analysis

Data were analyzed using Microsoft Excel and SPSS. Smoking prevalence on hospital grounds was calculated by the number of observed smokers as a proportion of all people observed during the observation period. To assess the impact of the SFP, we compared smoking prevalence on hospital grounds before and after implementation of the SFP. Independent samples t-tests were conducted to determine whether changes in smoking prevalence were significant. Bubble maps were created to visualize changes in geographical distribution of smokers at VUmc and AMC, before and after implementation of the SFP.

## RESULTS

Up to and including Table 2, the results presented in this section relate to the observations conducted 7 weeks before and 7 weeks after implementation of the SFP. The results of the follow-up observations, at 5 and 18 months afterwards, are reported subsequently.

**Table 1:** Number of smokers and smoking prevalence (%) before and after implementation of the SFP

*Site*	*Before implementation*			*After implementation*			p*
	*Number of people*	*Number of smokers*	*Smoking prevalence*	*Number of people*	*Number of smokers*	*Smoking prevalence*	
VUmc	1100	152	13.8	854	35	4.1	0.000
AMC	678	156	23.0	657	15	2.3	0.000
Total	1778	308	17.4	1500	50	3.3	0.000

* p-value for decrease in smoking prevalence (output of independent samples t-test).

**Table 2:** Number of observed smokers per category before and after implementation of the SFP

*Category*	*Before implementation*	*After implementation*	*Decrease (%)*
Staff	121	4	−96.7
Patients	39	3	−92.3
Students	20	14	−30.0
Visitors	128	29	−77.3
Total	308	50	−83.8

### Weather conditions

The mean temperature during the observations was 15.7°C (range: 6.8–23.4°C). The sun was shining (sometimes or continuously) during 42.9% of the observations while rain (varying from drizzle to more heavy rain) occurred during 25%. Although the sun was shining more often during the first observation rounds (in early Fall) and rain occurred more often during the final rounds (in late Fall), there were no substantial differences in weather conditions before and after implementation of the SFP.

### Number of smokers and smoking prevalence

Table 1 presents the number of smokers and smoking prevalence before and after implementation of the SFP, including differences between the two hospital locations. Before implementation of the SFP, a total number of 308 smokers were observed, with an average of 44 smokers per observation round (range: 35–54). After implementation of the SFP, a total number of 50 smokers were observed, with an average of 7 smokers per observation round (range: 1–12). Smoking prevalence on hospital grounds decreased significantly from 17.4% before to 3.3% after implementation of the SFP. The largest decrease in smoking prevalence was observed at location AMC (from 23.0% to 2.3%).

**Figure 1:**
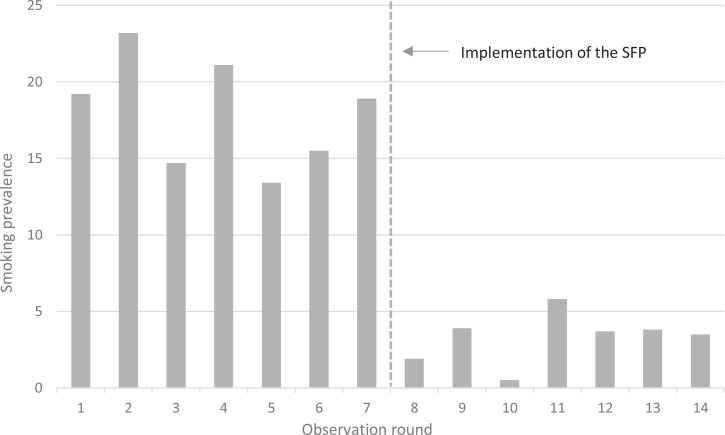
Smoking prevalence (%) on hospital grounds during each observation round

Figure 1 shows the smoking prevalence on hospital grounds during each of the 14 observation rounds (7 rounds before and 7 rounds after implementation). The dotted vertical line represents the implementation of the SFP on 1 October 2019. A sudden decrease in smoking prevalence immediately after implementation was observed.

### Number of smokers at each site

**Figure 2:**
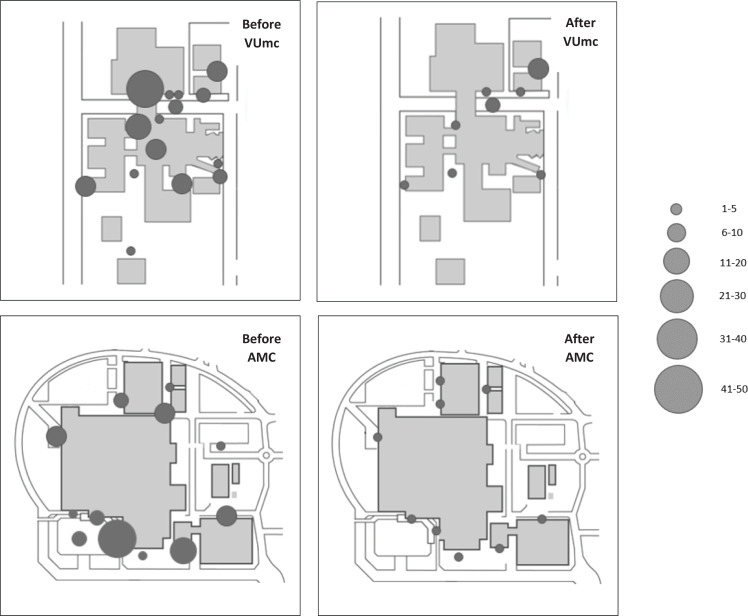
Bubble maps of observed smokers per site before (left) and after (right) implementation of the SFP

The bubble maps in Figure 2 show the number of observed smokers at each specific site before and after implementation of the SFP. At most of the sites, the number of smokers decreased after implementation of the SFP. Large decreases were observed around the entrances of the hospitals and outpatient clinics and where smoking shelters used to be present. At a number of sites, no smokers were observed after implementation of the SFP (indicated by ‘missing’ bubbles on the map). At VUmc, the number of smokers remained practically the same at sites that were not covered by the SFP (on the upper right side of the bubble map).

### Number of smokers per category

Table 2 presents the number of smokers per category before and after implementation of the SFP. Following implementation, the largest decrease in the number of smokers was observed among staff (-96.7%) and patients (-92.3%), while the smallest decrease was observed among students (-30.0%).

### Follow-up observations

Table 3 presents the smoking prevalence on hospital grounds during the follow-up observations at 5 and 18 months after implementation of the SFP. The decrease in smoking prevalence was found to be sustained (p=0.000). Between the two follow-up observations, a small, non-significant increase in the total smoking prevalence was reported (+2.4%), which could be attributed to a significant increase in smoking prevalence at location VUmc (+5.0%, p<0.05).

**Table 3:** Number of smokers and smoking prevalence (%) during follow-up observations

*Site*	*At 5 months*			*At 18 months*		
	*Number of people*	*Number of smokers*	*Smoking prevalence*	*Number of people*	*Number of smokers*	*Smoking prevalence*
VUmc	131	3	2.3	219	16	7.3
AMC	253	7	2.8	185	4	2.2
Total	384	10	2.6	404	20	5.0

## DISCUSSION

### Key findings

The aim of this study was to assess the impact of the SFP at the Amsterdam UMC on smoking prevalence on hospital grounds. After implementation of the SFP in 2019, smoking prevalence on hospital grounds decreased abruptly and significantly, with follow-up observations showing that this decrease was sustained. The largest decrease in the number of smokers was observed among staff and patients. The number of smokers decreased at most sites.

### Interpretation of findings

The substantial and sustained decrease in smoking prevalence and number of smokers found in this study highlights the potential of SFPs on hospital grounds to protect people from exposure to (secondhand) smoking. Similar results were reported by Poder et al.^8^ and Breunis et al.^10^.

The positive outcomes of the SFP found in our study may have resulted from the comprehensive and multifaceted preparation prior to the implementation of the SFP. It is likely that the numerous meetings with stakeholders have contributed to enhanced support for the SFP. Furthermore, much attention was paid to widespread communication of the SFP, such as through letters and signs, all of which used a positive tone. Previous studies have shown that enhancing support and positive communication are important determinants for successful implementation of SFPs^14,15^.

We found the largest decrease in the number of smokers among staff and patients. This may relate to the fact that smoking cessation support was offered to staff and nicotine replacement and behavioral therapy were available to admitted patients. Moreover, staff and patients may be more responsive to the SFP owing to the intensive advocacy within the hospital prior to implementation.

We found that smoking prevalence at location VUmc, but not at AMC, slightly increased between the follow-up observations in 2020 and 2021. This difference may be explained by the fact that in 2021 enforcement officers were present on the grounds of AMC, but not at VUmc. This suggests that the presence of enforcement officers can help ensure compliance with the SFP. Some previous studies have also shown that the use of enforcement officers fosters successful implementation of an SFP^16-19^. Enforcement does not necessarily need to be carried out by specially hired officers, as it was in our case, but can be incorporated into the roles of other staff such as security or general staff (e.g. nurses, doctors)^17,20^.

Compared to the studies of Poder et al.^9^ and Breunis et al.^10^, the decrease in the number of smokers was found to be larger in the current study. This may relate to the extent to which the SFP was actively enforced. For example, although in Rotterdam board members of the hospital and hired personnel were supposed to confront people who smoked, the study reported that very few smokers were actually asked to smoke elsewhere. The findings of the current study also indicate the importance of enforcement of the SFP.

Contrary to the general trend, the number of smokers at location VUmc remained almost the same at some sites. This mainly concerned the road between the hospital and the outpatient clinic, which was not covered by the SFP as it was considered municipal land. Studies in settings other than hospital grounds also reported that smokers tend to concentrate at adjacent places that are not covered by the SFP^14,21^. These findings emphasize the need to look at possibilities to further extend the reach of the SFP. For example, the municipality of Amsterdam is developing policies to discourage smoking within zones close to public buildings or smoke-free zones.

### Limitations

Three limitations need to be taken into account when interpreting the results. First, a relatively large number of smokers were categorized as ‘visitors’ because we were not always able to adequately categorize individuals. It is likely that this category also includes some staff, patients (including outpatients), and students who were not recognized as such. For example, a staff member may have left the hospital having changed clothes. Furthermore, patients (especially outpatients) do not always wear a hospital gown or wristband. Second, in a before-after design, one should be aware that secular trends or irregular changes might have caused the observed changes of interest^22^. However, we do not know of any external event that could have caused the large drop immediately after 1 October 2019. Third, observations were conducted once a week during a specific time frame (between 11:30 and 15:00). Although we expect that most smokers smoke during these hours (as it covers lunch break) and that our main results would have been the same if we choose different time frames, bias may have occurred.

### Implications

The current study has a number of implications. First, comprehensive preparation prior to the implementation of the SFP (including smoking cessation support) may enhance support and therefore compliance. Second, in order to ensure ongoing compliance, enforcement – either by enforcement officers or staff – is essential. Finally, hospitals and municipalities should cooperate in order to prevent smokers from concentrating just outside the smoke-free zones.

## CONCLUSIONS

This study highlights the potential of SFPs on hospital grounds to protect people from exposure to (secondhand) smoking. Continued enforcement of these SFPs seems essential to ensure ongoing compliance.

## Data Availability

The data supporting this research are available from the authors on reasonable request.
